# Prenatal Diagnosis and Management of Kaposiform Hemangioendothelioma With Kasabach–Merritt Phenomenon: Imaging Features and First Experience With Maternal Sirolimus Therapy

**DOI:** 10.1002/pd.70180

**Published:** 2026-05-16

**Authors:** Antoine Fraissenon, Chris Minella, Marion Bordas‐Fournel, Anne Gaëlle Grebille, Olivia Boccara, Marie Cassart, Sarah Simon‐Locatelli, Gwenaelle André, Guillaume Canaud, Alexandre Guilhem, Sara Cabet, Laurent Guibaud

**Affiliations:** ^1^ Centre de Référence des Anomalies Vasculaires Superficielles Imagerie pédiatrique et fœtale Hôpital Femme Mère Enfant Université Claude Bernard Lyon 1 Villeurbanne France; ^2^ INSERM U1151 « Mécanismes et stratégies thérapeutiques dans les syndromes de surcroissance et les anomalies vasculaires » Institut Necker Enfants Malades Paris France; ^3^ Service de Radiologie Mère‐Enfant Hôpital Nord CHU Saint Etienne Saint‐Priest‐en‐Jarez France; ^4^ Centre de Diagnostic Prénatal Hôpitaux de Strasbourg Strasbourg France; ^5^ Centre de Diagnostic Prénatal Hôpital de Saint Brieux Saint‐Brieuc France; ^6^ Dermatologie Pédiatrique Hôpital Necker Paris France; ^7^ Department of Radiology and Fetal Medicine Iris Hospitals South Brussels Belgium; ^8^ Réanimation néonatale Hôpitaux de Strasbourg Strasbourg France; ^9^ Centre de Diagnostic Prénatal CHU de Bordeaux Bordeaux France

**Keywords:** fetal MRI, fetal tumor, kaposiform hemangioendothelioma, kasabach–merritt phenomenon, maternal sirolimus therapy, prenatal ultrasound, sirolimus, vascular anomalies

## Abstract

**Objective:**

To describe the prenatal diagnosis, evolution, and perinatal management of kaposiform hemangioendothelioma (KHE) complicated by the Kasabach–Merritt phenomenon (KMP), and to report the first documented use of maternal sirolimus therapy (MST) in this setting.

**Methods:**

We retrospectively reviewed four fetuses with a prenatal soft‐tissue mass suspected for KHE. Imaging characteristics, fetal complications, perinatal outcomes, and postnatal hematologic data were analyzed. MST was initiated in one severe case with rapidly progressive disease and early fetal compromise.

**Results:**

All lesions demonstrated infiltrative margins, marked hypervascularity, and T2‐hyperintense signal on MRI. Three fetuses showed prenatal signs suggestive of evolving KMP. Two pregnancies were terminated. Among the two liveborn infants, one developed early neonatal KMP requiring urgent sirolimus therapy. All lesions demonstrated infiltrative margins, marked hypervascularity, and T2‐hyperintense signal on MRI. Three fetuses showed prenatal signs suggestive of evolving KMP. Two pregnancies were terminated. Among the two liveborn infants, one developed early neonatal KMP requiring urgent sirolimus therapy. In the most recent case, a temporal association was observed between MST initiation and early reduction of tumor infiltration on follow‐up MRI. The infant was delivered at 39 weeks and responded to combined sirolimus–steroid therapy.

**Conclusion:**

Prenatal imaging can strongly suggest KHE and identify early signs of KMP. This series provides preliminary evidence that MST may represent a promising antenatal therapeutic option in selected severe cases.

## Summary

- What's already known about this topic?
  ◦ Kaposiform hemangioendothelioma (KHE) is a rare fetal tumor with high morbidity when complicated by the Kasabach–Merritt phenomenon. Prenatal diagnosis is exceptional, but characteristic imaging features exist. Maternal sirolimus therapy (MST) has never been used for fetal KHE.
- What does this study add?
  ◦ This series details prenatal KHE features, identifies signs of evolving KMP, and reports the first prenatal MST use.

## 1 Introduction

Kaposiform hemangioendothelioma (KHE) is a rare infiltrative vascular tumor of infancy, and its most severe complication—Kasabach–Merritt phenomenon (KMP)—is associated with substantial morbidity and mortality due to profound consumptive coagulopathy [1, 2]. Prenatal diagnosis of KHE remains exceptional, but advances in fetal imaging have increased the recognition of infiltrative soft‐tissue masses suggestive of this entity.

Identifying KMP in utero is particularly challenging, as laboratory confirmation is rarely feasible before birth. Nevertheless, several reports, including one from our group, indicate that certain imaging and clinical features may suggest evolving coagulopathy [3, 4, 5]. Despite these emerging observations, data on prenatal presentation, evolution, and perinatal outcomes of KHE remain extremely limited, and no consensus exists regarding optimal antenatal management.

Therapeutic progress in the postnatal setting is therefore highly relevant. Sirolimus has become a cornerstone treatment for postnatal KHE complicated by KMP and has demonstrated significant efficacy in lymphatic malformations through inhibition of the PI3K/AKT/mTOR pathway [6, 7]. More recently, maternal sirolimus therapy (MST) has emerged as a promising prenatal option for severe fetal lymphatic malformations, with consistent transplacental drug transfer and encouraging fetal responses [8, 9, 10, 11]. Whether MST could benefit fetuses with rapidly progressive KHE at risk of KMP has not yet been systematically explored.

In this context, we present a 19‐year retrospective series of prenatally detected KHE, describe the imaging features that support antenatal diagnosis, and report the first documented use of MST in a fetus with rapidly progressive KHE and early signs of KMP.

## 2 Material and Methods

We conducted a retrospective review of all fetuses referred to our National Reference Center between January 2006 and January 2025 for the evaluation of a suspected vascular soft‐tissue lesion. Eligible cases met two criteria: [1] identification of a mass on prenatal ultrasound and/or MRI, and [2] postmortem or postnatal confirmation of kaposiform hemangioendothelioma (KHE). In Cases 1 and 2, the diagnosis was established by postmortem pathological examination following termination of pregnancy. In Case 3, histopathological confirmation was obtained postnatally. In Case 4, which received prenatal maternal sirolimus therapy, the diagnosis relied on characteristic imaging features, clinical evolution, and postnatal course consistent with KHE, without biopsy. Histologically confirmed cases are indicated in Table 1.

**TABLE 1:** Prenatal characteristics of the four fetuses diagnosed with kaposiform hemangioendothelioma (KHE).

Case	GA at diagnosis	Location	Growth	Doppler hypervascularity	Infiltrative T2W MRI	Fetal cardiac decompensation/anemia	Suspected KMP
1	25 w	Lower limb	Rapid	Marked	Marked	C, A, P	Yes
2	33 w	Thoracic wall	Rapid	Marked	Marked	No	Yes
3	25 w	Face	No	Discrete	Marked	No	No
4	34 w	Face	Rapid	Marked	Marked	H (pleural effusion)	Yes

*Note:* Histopathological confirmation: Cases 1 and 2 (postmortem); Case 3 (postnatal biopsy). Case 4 diagnosed based on imaging and clinical evolution.

Abbreviations: A = anemia; C = cardiomegaly; H = hydrops/effusions; P = polyhydramnios.

All available prenatal imaging studies were reviewed. Ultrasound examinations were assessed for lesion size, echotexture, internal architecture, depth of subcutaneous and deep‐tissue infiltration, and vascularity on color or power Doppler. Prenatal suspicion of KHE relied on the presence of an infiltrative heterogeneous echogenic soft‐tissue mass with marked subcutaneous thickening, pronounced hypervascularity, and rapid interval growth. When performed, fetal MRI was analyzed for T2‐hyperintense signal, infiltrative margins, and extension into fascia, muscle compartments, or adjacent soft‐tissue planes, using T2‐weighted and T1‐weighted sequences. Serial ultrasound and MRI examinations were used to evaluate interval growth and associated findings.

Clinical data were extracted from maternal and fetal records, including gestational age at diagnosis, presence of [fetal cardiac decompensation and/or fetal anemia], polyhydramnios, cardiomegaly, skin edema, effusions, and imaging features suggestive of Kasabach–Merritt phenomenon (KMP). Perinatal management strategies were documented, including decisions regarding pregnancy continuation or termination, timing and mode of delivery, and use of prenatal and/or postnatal anti‐angiogenic therapy.

For liveborn infants, neonatal laboratory parameters, treatment protocols, and clinical outcomes were reviewed. In the most recent case treated with maternal sirolimus therapy (MST), maternal tolerance, dosing strategy, therapeutic monitoring, and fetal response were specifically analyzed. Ethical approval for retrospective analysis of anonymized clinical data was obtained in accordance with institutional guidelines.

## 3 Results

**FIGURE 1:**
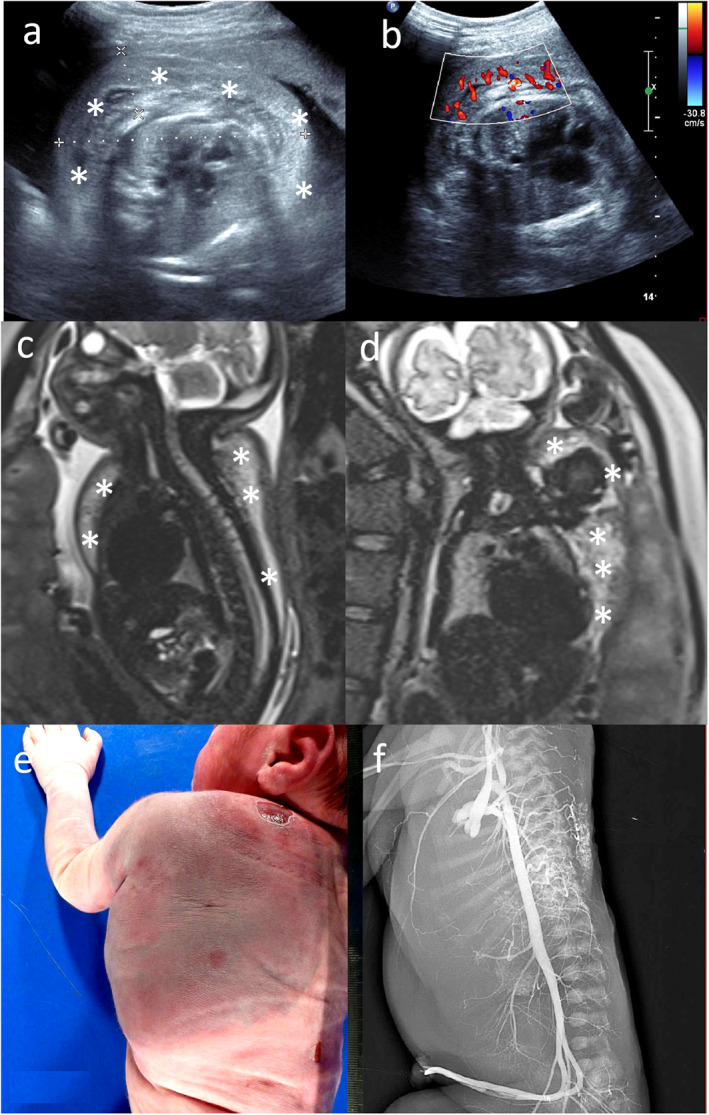
(a–f, Case 2). Axial Doppler‐ultrasound images at 35 weeks' gestation show an echogenic, markedly hypervascular infiltrative mass of the left thoracic wall, involving both subcutaneous tissues and the intercostal musculature (a, b). These findings correspond to the T2‐hyperintense infiltrative lesion seen on midsagittal and coronal fetal MRI, which better delineates the extent of deep‐tissue involvement (c, d). Given the severity of the lesion, the parents elected termination of pregnancy. Macroscopic examination confirmed the prenatal imaging features and demonstrated the characteristic violaceous skin discoloration of KHE (e). Postmortem angiography via the umbilical artery revealed arterial supply from multiple intercostal branches arising from the thoracic aorta (f).

**FIGURE 2:**
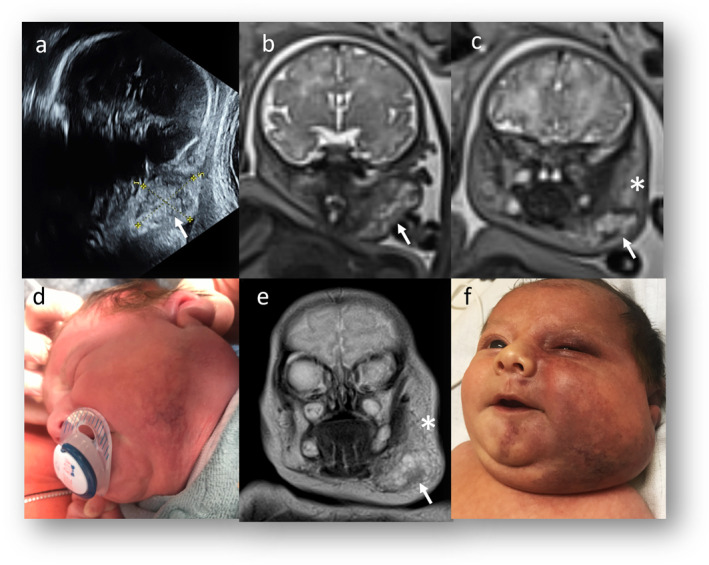
(a–f, Case 3). Coronal Doppler ultrasound at 33 weeks' gestation shows an echogenic infiltrative mass involving the left subcutaneous and retromandibular soft tissues (a, arrow). These findings correspond to the T2‐hyperintense infiltrative lesion seen on coronal fetal MRI (b, c, arrow), with discrete involvement of the left temporal soft tissues compared with the normal right side (c). Clinical examination at birth confirmed the prenatal imaging findings and demonstrated violaceous angiomatous discoloration of the overlying skin (d). Postnatal coronal T2‐weighted MRI showed stable discrete hyperintensity of the left submandibular and temporal infiltration (e, arrow). During the first two weeks of life, the infant developed significant clinical deterioration with increased facial infiltration and progressive violaceous discoloration, associated with thrombocytopenia (26 G/L), hypofibrinogenemia (1 g/L), and markedly elevated D‐dimer levels (> 20,000 ng/mL). KMP was diagnosed based on these characteristic findings, prompting the emergency initiation of combined sirolimus–steroid therapy (f).

**FIGURE 3:**
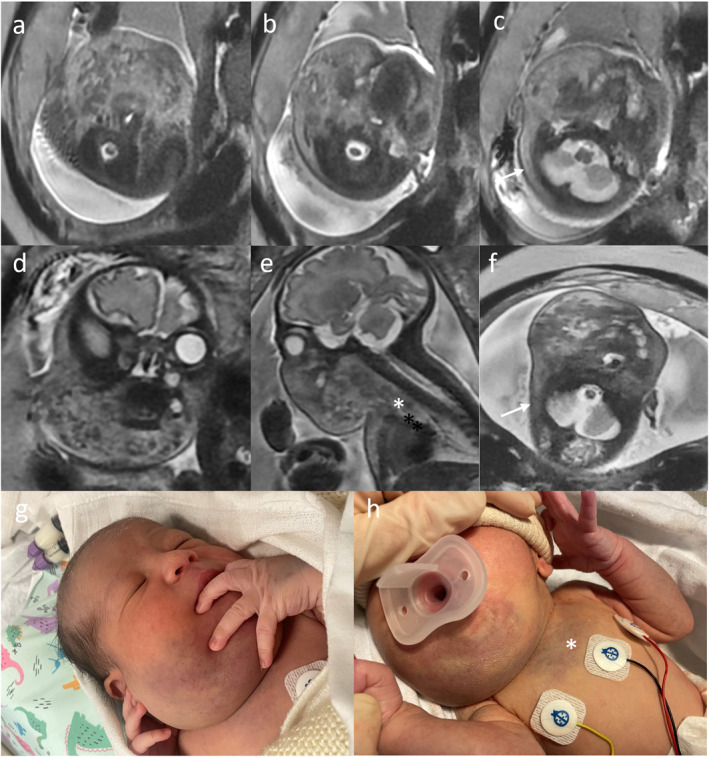
(a–h, Case 4). Axial and coronal T2‐weighted fetal MRI at 32 weeks' gestation (a, d) shows a large bilateral submandibular hyperintense infiltrative lesion involving both subcutaneous and deep soft tissues of the lower face (sublingual and right parotid regions), with right‐sided predominance and extension into the right posterior cervical tissues (c, arrow). Additional anterior thoracic wall involvement is visible on sagittal MRI (e, *). Follow‐up axial MRI 3 weeks after initiation of maternal sirolimus therapy demonstrates decreased extent of infiltration and reduced T2‐signal intensity in the right cheek and right posterior cervical tissues (f). Clinical examination at birth confirmed right lower‐face (g) and anterior thoracic wall infiltration (h), with violaceous skin discoloration that was less marked than in Figure 2f at the onset of KMP. Laboratory tests showed thrombocytopenia (30 G/L), consistent with ongoing KMP.

Four fetuses were diagnosed prenatally with a soft‐tissue mass suspicious for KHE: one in the second trimester (25 weeks) and three in the late third trimester (≥ 32 weeks). One fetus presented with a lower‐limb lesion, a case previously reported by our group [5], while the remaining three involved the thoracic wall (*n* = 1; Figure 1a–f, Case 2) and the face (*n* = 2; Figure 2a–f, Case 3; Figure 3a–h, Case 4).

All lesions except one demonstrated rapid growth. Ultrasound consistently showed a heterogeneous echogenic mass with marked subcutaneous thickening and pronounced hypervascularity. Fetal MRI confirmed infiltrative T2‐hyperintense lesions in all cases, with deep cervicofacial and anterior thoracic extension in the most recent case (Figure 3d,e, Case 4).

Signs of fetal cardiac decompensation and/or fetal anemia were present in three fetuses, including polyhydramnios, cardiomegaly, and early pleural effusion. Based on rapid tumor progression and these associated findings, KMP was suspected prenatally in three cases and developed postnatally in the fourth.

Two pregnancies were terminated because of poor prognosis, including one diagnosed at 25 weeks with rapid tumor progression. Among the two liveborn infants, one developed KMP on day 1 of life, characterized by abrupt inflammatory changes of the lesion (Figure 2f, Case 3) and a sudden platelet drop, prompting immediate neonatal sirolimus therapy with subsequent hematologic improvement.

In the most recent case, prenatal ultrasound and MRI demonstrated a rapidly enlarging infiltrative cervicofacial mass extending toward the anterior thoracic soft tissues (Figure 3a–e, Case 4) associated with early pleural effusion—findings strongly suggestive of KHE with evolving KMP. Given the high risk of fetal demise and supported by our experience with sirolimus in KMP [6] and with maternal sirolimus therapy (MST) for extensive lymphatic malformations [8, 9, 10, 11, 12], MST was initiated at 34 weeks following multidisciplinary counseling and written informed maternal consent. The mother weighed 64 kg (BMI 23 kg/m^2^) at the beginning of pregnancy and 80 kg (BMI 28 kg/m^2^) at treatment initiation. Sirolimus was administered once daily, starting at 8 mg/day and escalated to 10 mg/day to achieve target trough levels, consistent with targets established for MST in extensive lymphatic malformations [12]. Maternal monitoring included lipid panel, liver function tests, fasting glucose, blood pressure, and clinical assessment for mucositis and infection; no adverse effects were observed. Weekly ultrasound was performed to detect any emerging signs of fetal cardiac decompensation and/or fetal anemia.

Follow‐up fetal MRI performed 3 weeks after MST initiation showed early reduction in T2‐weighted signal infiltration (Figure 3f, Case 4). Sirolimus levels at delivery were 12.3 ng/mL in umbilical cord blood and 11 ng/mL in the newborn, confirming effective transplacental transfer.

A male infant was delivered at 39 weeks by elective caesarean section with excellent neonatal adaptation. Postnatal clinical examination confirmed extensive KHE involving the anterior thoracic wall, right submandibular region, and lower right face, with characteristic violaceous skin discoloration. Postnatal MRI further delineated the extent of infiltration (Figure 3g,h, Case 4). Laboratory evaluation demonstrated KMP, with severe thrombocytopenia (30 G/L) and markedly elevated D‐dimer levels. Oral sirolimus was initiated on day 1 of life, and aspirin was added on day 3. Exclusive maternal breastfeeding was performed, although sirolimus excretion in breast milk warrants caution, particularly in the context of concurrent neonatal sirolimus therapy.

Combined postnatal sirolimus and corticosteroid therapy resulted in progressive hematologic improvement within 3 weeks and complete normalization of platelet count by 6 weeks, allowing discharge home on continued sirolimus therapy. Maternal postpartum follow‐up was uneventful.

Prenatal characteristics and postnatal outcomes of the four cases are summarized in Tables 1 and 2.

**TABLE 2:** Postnatal outcomes, hematologic findings, treatment, and follow‐up.

Case	GA at delivery	Birth	Platelets	Fibrinogen	Max lesion size	NICU stay/Ventilation	Treatment & follow‐up
1	N/A (TOP)	TOP	—	—	N/A	N/A	N/A
2	N/A (TOP)	TOP	—	—	Entire thoracic wall	N/A	N/A
3	38 w	Liveborn	Normal → 26 G/L	1 g/L (KMP)	∼5 cm (left submandibular)	21 days/No ventilation	Sirolimus + steroids; platelets normalized at 6 weeks; sirolimus continued 18 months
4	39 w	Liveborn	30 G/L	Low	∼8 cm (cervicofacial + thoracic)	42 days/No ventilation	Prenatal MST (10 mg/day); postnatal sirolimus + steroids + aspirin (day 3); platelets normalized at 6 weeks; discharged on sirolimus

Abbreviations: MST = maternal sirolimus therapy; NICU = neonatal intensive care unit; TOP = termination of pregnancy.

## 4 Discussion

This study provides one of the most comprehensive prenatal descriptions of kaposiform hemangioendothelioma (KHE) to date and underscores the importance of recognizing its characteristic imaging features in utero. Although KHE is typically diagnosed postnatally based on clinical, biological, and imaging findings, advances in fetal imaging have facilitated increasing identification of this entity before birth. In our series, the combination of tumor growth, infiltrative margins, hypervascularity, and T2‐hyperintense signal on MRI consistently supported the antenatal suspicion of KHE. These features—particularly the infiltrative pattern extending into subcutaneous tissues and muscle compartments—help distinguish KHE from congenital hemangiomas, which are usually well circumscribed. Given the extreme rarity of prenatal KHE, the reproducibility of these imaging criteria across cases is noteworthy and may assist clinicians in refining antenatal diagnosis.

Kasabach–Merritt phenomenon (KMP) remains the most critical determinant of prognosis in KHE. Its pathophysiology—platelet trapping, consumptive coagulopathy, and microangiopathic hemolysis—can evolve rapidly in utero, leading to fetal cardiac decompensation and/or fetal anemia, hydrops, and intrauterine demise. Although laboratory confirmation of KMP is rarely feasible before birth, our findings suggest that a constellation of imaging and clinical features may serve as early warning signs for fetal coagulopathy. In our series, indirect signs such as rapid tumor enlargement, increasing vascularity, cardiomegaly, polyhydramnios, and early effusions were strongly suggestive of evolving KMP. These observations are consistent with previous reports, including the case series by Beissel et al. [5].

Regarding the potential role of fetal blood sampling, this procedure could theoretically confirm KMP by demonstrating thrombocytopenia and hypofibrinogenemia in utero. However, given the risk of haemorrhagic complications in the context of consumptive coagulopathy, and the technical challenges associated with fetal blood sampling in the third trimester, its routine use cannot be recommended. The indirect imaging signs described above appear sufficient to suspect KMP and guide clinical decision‐making in most cases.

Over the past decade, sirolimus has transformed both the postnatal management of KHE and the treatment of lymphatic malformations (LM), particularly extensive microcystic or mixed lesions. Multiple pediatric and neonatal studies have demonstrated rapid clinical improvement and volumetric reduction under sirolimus therapy, mediated through inhibition of the PI3K/AKT/mTOR pathway [6, 7]. Although KHE and LM differ histologically, both share dysregulated angiogenic and lymphangiogenic signaling [6, 13], providing a biologically plausible rationale for therapeutic overlap.

Recent reports of maternal sirolimus therapy (MST) for severe fetal LM have demonstrated the feasibility of prenatal targeted therapy, with consistent transplacental drug transfer, acceptable maternal tolerance, and encouraging perinatal outcomes [8, 9, 10, 11]. Building on this emerging experience, our most recent case suggests that MST may also play a role in selected cases of fetal KHE complicated by evolving KMP. In this context, MST was initiated using an initial maternal dose of 8 mg/day and escalated to 10 mg/day to achieve a trough level of 10–15 ng/mL—a target derived from our experience with MST in extensive LM and supported by emerging pharmacokinetic data indicating that maternal levels in this range are required to ensure adequate fetal exposure [12].

Regarding maternal safety, the pre‐therapeutic assessment included complete blood count, renal and hepatic function, fasting lipid panel, fasting glucose, blood pressure, and screening for active infection. During treatment, regular clinical and biological monitoring was performed. In our case, no adverse effect was observed during the treatment period, and the postpartum course was equally uneventful. However, known risks of sirolimus in the maternal context include dyslipidaemia, impaired wound healing (particularly relevant in the context of caesarean delivery), increased susceptibility to infections, mucositis, hypertension, and glucose intolerance, and systematic evaluation of maternal safety remains essential. Regarding breastfeeding, exclusive maternal breastfeeding was performed and encouraged in this case. However, given that sirolimus is excreted in breast milk, caution is warranted: the potential systemic neonatal exposure—particularly when the infant is simultaneously receiving postnatal sirolimus therapy—requires careful individual benefit–risk assessment.

A temporal association between MST initiation and reduced T2‐weighted infiltration on fetal MRI was observed. However, causality cannot be formally established from a single case without a control group, especially given the short interval between treatment initiation and follow‐up imaging. Spontaneous changes, measurement variability, or fluctuations in tissue edema cannot be excluded. Nevertheless, the clear and rapid postnatal response to sirolimus in this infant—mirroring the antenatal imaging improvement—and the disappearance at birth of the prenatal signs of fetal compromise, including complete resolution of the pleural effusion with a clinically well newborn, strongly support the likelihood that the prenatal changes were treatment‐related rather than incidental. This concordance between prenatal and postnatal evolution reinforces the hypothesis that MST may have contributed to early modulation of tumor activity, while still warranting confirmation in prospective studies.

Given the potential for abrupt clinical deterioration in fetal KHE—including sudden hemorrhage, hydrops, or intrauterine demise—prenatal discussion of MST may be warranted even in the absence of overt fetal cardiac decompensation and/or fetal anemia, particularly when rapid tumor progression or early signs of KMP are present. However, MST should not be considered a universal strategy; rather, it may represent a targeted option within a multidisciplinary decision‐making framework reserved for the most severe and rapidly progressive cases.

Several limitations of this study should be acknowledged. First, the retrospective design limits the level of evidence. Second, as a national reference center, our series is enriched for severe phenotypes; mild or misdiagnosed cases may have been referred elsewhere or remained undetected. Third, the small sample size (*n* = 4) precludes any statistical inference or generalization of the findings. Fourth, Case 4 was not histologically confirmed, reflecting the clinical reality that biopsy is rarely feasible in the prenatal context.

Despite these limitations, our findings contribute to a growing body of evidence supporting the feasibility of prenatal targeted therapy in selected severe vascular anomalies. Further structured prospective studies are now needed to clarify its efficacy, refine dosing strategies, and define its place within the antenatal management algorithm of fetal vascular tumors.

## Funding

The authors have nothing to report.

## Ethics Statement

Institutional Review Board CERIM 16^th^ July 2025, CRM‐2507‐483.

## Conflicts of Interest

The authors declare no conflicts of interest.

## Data Availability

The data that support the findings of this study are available from the corresponding author upon reasonable request.
